# Prevalence of IgE‐mediated sensitization in patients with suspected food allergic reactions in Jordan

**DOI:** 10.1002/iid3.320

**Published:** 2020-06-15

**Authors:** Issa Abu‐Dayyeh, Jumana Abu‐Kwaik, Alf Weimann, Amid Abdelnour

**Affiliations:** ^1^ Biolab Diagnostic Laboratories Amman Jordan; ^2^ Department of Basic Research, Cell Therapy Center University of Jordan Amman Jordan; ^3^ Euroimmun AG Lübeck Germany

**Keywords:** epidemiological study, food allergy, food sensitization, Jordan, specific IgE test

## Abstract

**Background:**

Despite the recognition of food allergies as a significant cause of morbidity and a growing public health burden worldwide, there are no epidemiological studies addressing food sensitization and allergy in Jordan.

**Objective:**

To conduct an epidemiological study that retrospectively analyzes sensitization data from 3463 patients with suspected immunoglobulin E (IgE)‐mediated food allergic reactions who performed specific IgE tests in our laboratories in Jordan.

**Methods:**

Specific IgE (s‐IgE) tests were analyzed for patients who performed enzyme allegro‐sorbent testing based on either self‐reported food allergy or upon physician's request.

**Results:**

2.3% of the analyzed samples were cross‐reactive to carbohydrate determinants. A quarter of the patients were sensitized to one or more food allergens, with males having higher odds of being s‐IgE positive. Furthermore, a higher prevalence of sensitization was seen in infants and children compared with adults. s‐IgE was most frequently found against cow milk (11.2%), pistachio (4.9%), soybean (4.6%), cherry (4.4%), and orange (4.4%). Interestingly, the s‐IgE class distribution profile of pistachio differed from the rest of the top hits being skewed away from the weak class 1 leaning more towards higher IgE classes. Food allergen sensitization was age group‐dependent: milk, tree nuts, and eggs were the main food groups causing sensitization in infants, while it was fruits followed by milk in children and adults.

**Conclusions:**

Our work represents the first epidemiological study addressing food sensitization in Jordan. This study lays a solid foundation for future studies that can help better guide food allergy diagnosis, patient dietary modifications, and food elimination plans, as well as assist decision‐makers in the region to develop national strategies for an efficient and sustainable healthcare system.

## Graphical abstract

This is the first epidemiological study to investigate the prevalence of food sensitization in Jordan, and one of very few in the Middle East region. We report food‐specific immunoglobulin E (IgE) sensitization and carbohydrate determinant prevalence to a large number of food items in a sizeable and representative cohort of patients suspected of IgE‐mediated food allergic reactions in Jordan. This study reveals gender and age group‐dependent differences in the prevalence of food IgE sensitization, as well as a unique s‐IgE class distribution profile for pistachio, being skewed towards higher IgE classes.

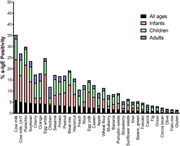

## 1 INTRODUCTION

Food is an important physiological requirement for life. Nevertheless, ingested food may cause adverse immunological responses, especially to protein elements.^1^ Food sensitization (FS) is a preliminary step to developing food allergy (FA). Sensitization usually begins in infancy and is achieved by the production of specific immunoglobulin E (s‐IgE) antibodies against certain foods.^2^ B‐cells are responsible for the production of food s‐IgE antibodies which, in turn, have the ability to bind specific receptors on tissue mast cells, therefore arming them. Upon reexposure to the triggering food allergen, armed mast cells secrete substances such as histamines, leukotrienes, and prostaglandins, all of which play important roles in triggering allergic reactions.^3^, ^4^, ^5^

FA incidence is increasing worldwide.^6^ Allergic reactions to food components remain an important cause of morbidity responsible for a significant proportion of severe reactions such as anaphylactic shock, which can be life‐threatening.^7^, ^8^ Enzyme allergo‐sorbent tests (EAST) measuring food s‐IgE antibodies in patient sera is a safe and useful tool for the identification of individuals who have an increased likelihood of reacting to certain foods and; therefore, developing clinical signs and symptoms of FA.^9^

There are no epidemiological studies regarding FS and FA in Jordan, and data on the prevalence of FA in the Middle East is fragmented and limited.^10^, ^11^, ^12^, ^13^, ^14^, ^15^ Therefore, evidence‐based efforts to reduce FA morbidity are greatly hindered by the scarcity/lack of available data, further complicating the national efforts in diagnosing, managing, and devising long‐term health plans concerning food allergies in the region.

In this manuscript, data collected retrospectively over the span of 5 years (August 2014‐August 2019) about the age, gender, and s‐IgE FS of patients in Jordan were analyzed. This study is the first epidemiological study coming from Jordan regarding IgE sensitization to a wide range of allergens belonging to foods consumed in the country.

## 2 MATERIALS AND METHODS

### 2.1 Food allergen s‐IgE test

Food allergen s‐IgE levels were determined semi‐quantitatively via in vitro EAST, using the IVD‐approved and CE‐certified EUROLINE immunoblot strip “Food Turkey 1” (Catalog #DP 3420‐1601‐11 E; Euroimmun, Lübeck, Germany; Figure S1). All strips were incubated in a semi‐automated fashion using EUROBlotMaster (Euroimmun) according to manufacturer's instructions. Briefly, test strips coated with 34 food allergens (egg white [f1], egg yolk [f75], cow milk [f2], cow milk ultra‐high temperature [UHT] [f169], casein [f78], wheat flour [f4], gluten [f79], rice [f9], soybean [f14], sesame [f10], peanut [f13], hazelnut [f17], pistachio [f144], sunflower seed [u87], pumpkin seed [f222], cacao [f73], orange [f33], strawberry [f44], apple [f49], banana [f92], kiwi [f84], mulberry [f146], fig [f328], tomato [f25], carrot [f31], potato [f35], onion [f48], peach [f95], cherry [f97], olive [f122], green bean [f132], fish mix 1 [f3, f21, f206, f254], meat mix 2 [fs43], chicken [f83]), in addition to a cross‐reactive carbohydrate determinant (CCD) and an indicator band were moistened and then incubated with 400 µL of undiluted patient serum to bind s‐IgE antibodies present. Subsequently, the bound s‐IgE antibodies were detected using an enzyme‐linked anti‐human IgE catalysing a color reaction. Strips were scanned and then analyzed for EAST class using EUROLineScan software (Euroimmun). A negative result where no s‐IgE is detected was denoted by class 0, while positive results were denoted by class 1 to 6, with low levels classified as class 1 and extremely high levels as class 6.

### 2.2 Patient data and inclusion/exclusion criteria

In accordance with the ethical approval number IRB/7/2019 issued by the Cell Therapy Center (CTC), existing patient‐related data used in this study, namely gender, age, and s‐IgE test results were collected retrospectively from Biolab Diagnostic laboratory's laboratory information management system (LIMS), in such a manner that participants cannot be identified directly or through any identifiers linked to them. Inclusion criteria involved patients of both sexes and all ages with a suggestive history of IgE‐mediated FA, tested for s‐IgE upon their request (69%), or that of their treating physician (31%), during the period from August 2014 to August 2019. Samples were obtained from patients who either physically presented to any of the 18 Biolab Diagnostic Laboratory branches in Jordan (16 branches in the capital Amman, 1 in the city of Fuheis [20 km northwest of Amman], and 1 in the city of Irbid [70 km north of Amman]), or presented to any non‐Biolab laboratory in Jordan that refers blood samples for s‐IgE testing to Biolab's immunology department (total of 62 laboratories, 51 of which are located in the capital Amman [central region], 6 laboratories in the cities of Irbid, Zarqa, and Jerash [northern region], and 5 laboratories in the cities of Karak, Tafilah, Ma'an, and Aqaba [southern region]). All CCD‐positive patients were excluded from the study (83 CCD‐positive tests out of 3647 tests performed during the indicated study period).

### 2.3 Graphs and statistical analyses

All graphs and statistical analyses were performed using GraphPad Prism (Version 8.0.2), 2019. Significance of the correlation between gender and IgE positivity was tested using contingency table analyses (*χ* ^2^ goodness of fit test with Yates' correction). The significance of the difference in IgE positivity between age groups was calculated using a one‐way analysis of variance followed by multiple comparisons. *P* less than .05 and lower were deemed statistically significant.

## 3 RESULTS

### 3.1 Characterizing the total and s‐IgE‐sensitized populations by gender and age group

**Figure 1:**
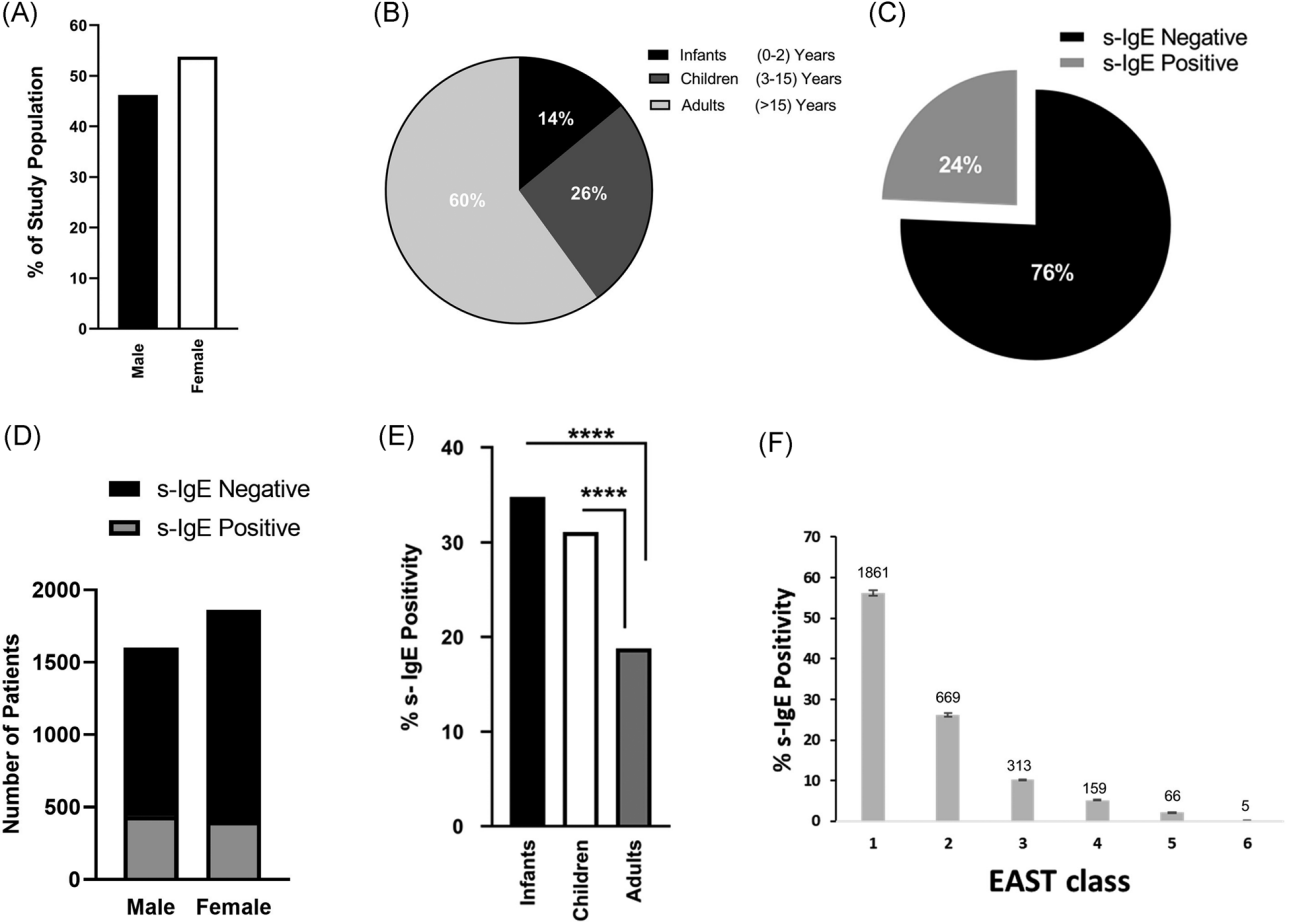
Characteristics of total and specific immunoglobulin E (s‐IgE)‐sensitized populations. A, Bar graph showing the sex distribution of the total study population. B, Pie chart displaying the distribution of the defined age groups of the total study population. C, Pie chart displaying the percentage of IgE‐sensitized patients (gray) out of the total study population. D, Bar graph representation of contingency table data. Each bar represents one gender and is divided based on the presence or absence of food s‐IgE antibodies in patient sera. Odds ratio = 1.357, *P* = .0001, 95% confidence interval = 1.16‐1.59. E, Bar graph comparing age group distribution of the s‐IgE‐positive subpopulation (****P < .0001). F, Bar graph displaying the s‐IgE class distribution of the s‐IgE‐positive subpopulation. Percentage class distribution for all positive food allergens was reported; the total positive count of each class is affixed on top of its respective bar. Class categories (kU/L): class 1 (0.35 ≤ s‐IgE < 0.70), class 2 (0.70 ≤ s‐IgE < 3.50), class 3 (3.50 ≤ s‐IgE < 17.50), class 4 (17.50 ≤ s‐IgE < 50.00), class 5 (50.00 ≤ s‐IgE < 100.00), class 6 (>100.00). EAST, enzyme allergo‐sorbent test

From a total of 3647 samples initially investigated, 3564 s‐IgE tests belonging to 3463 patients were included in the study and analyzed for gender and age group distribution. Results indicated that 46.2% of the patients were males (age range: 9 days‐94 years, mean age: 21.9 years), while 53.8% were females (age range: 1 day‐89 years, mean age: 27.5 years; Figure 1A). Furthermore, 14% of the patients were in the infant age group (0‐2 years, mean = 1.5 years), 26% were in the children age group (3‐15 years, mean = 6.9 years), while the majority of patients (60%) were in the adult group (>15 years, mean = 34 years; Figure 1B). Next, we aimed to find out how many patients displayed s‐IgE sensitization to any of the food allergens found in the immunoblot panel. Results indicated that around a quarter of the patients included in the study were sensitized to one or more food allergens (Figure 1C). Interestingly, the *χ* ^2^ test revealed a significant correlation between gender and s‐IgE positivity, with males having higher odds of being s‐IgE positive (odds ratio = 1.357; *P* = .0001; 95% confidence interval = 1.16‐1.59; Figure 1D). In terms of age groups, a significantly higher percentage of s‐IgE positive samples was reported in infants (34.8%) and children (31.1%) compared with adults (18.8%; Figure 1E). When arranging the semi‐quantitative values of the s‐IgE‐positive subpopulation by class, it was apparent that the majority fell in the range of class 1, and as the class value increased, the percentage of s‐IgE reported decreased (Figure 1F).

### 3.2 Prevalence of s‐IgE sensitization to the various food allergens

**Figure 2:**
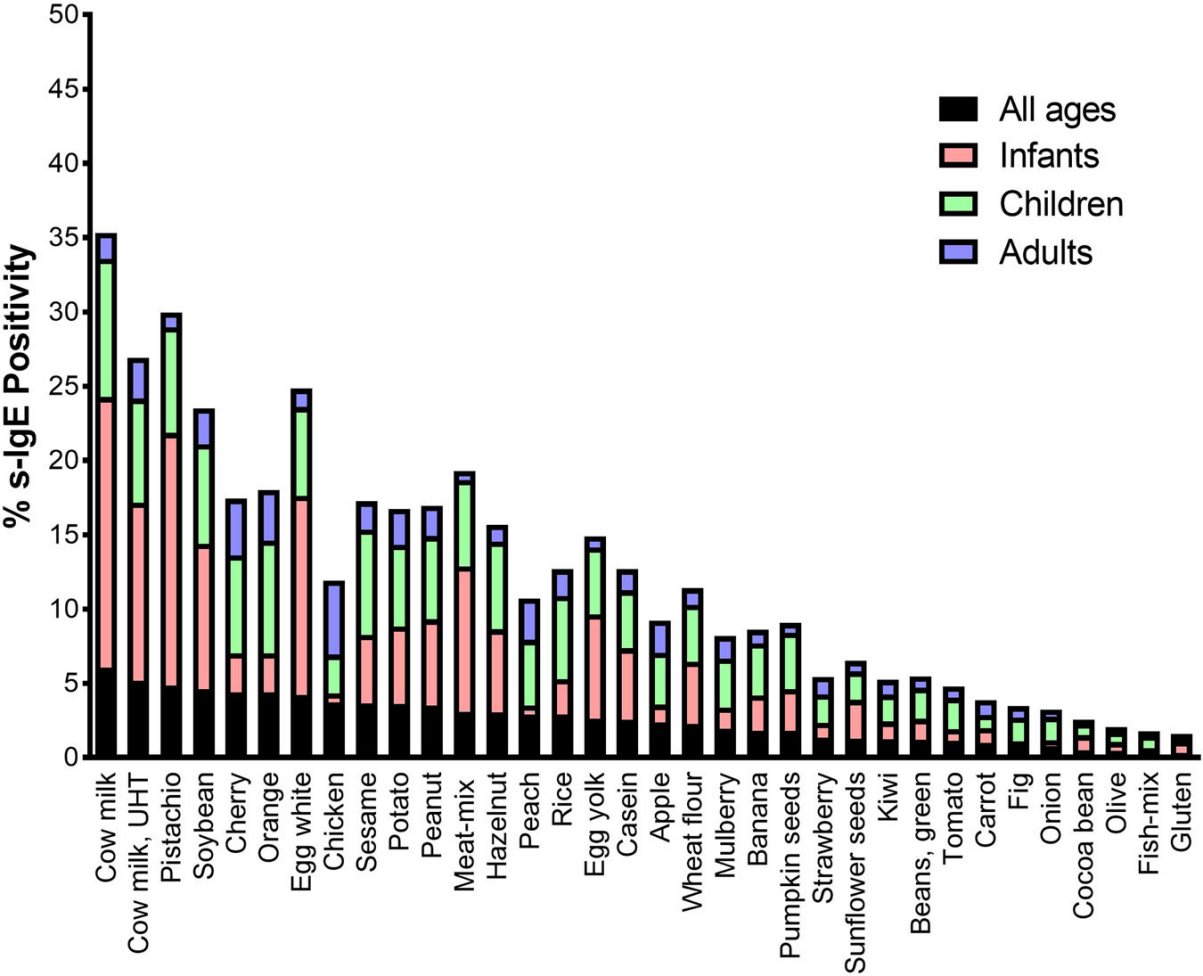
Prevalence of food‐specific immunoglobulin E (s‐IgE) antibodies in all sensitized patients combined and by age group. Stacked bar chart displaying the prevalence (%) of s‐IgE sensitization to the various food allergens for all sensitized patients combined (black stacks), infants; 0 to 2 years (pink), children; 3 to 15 years (green), and adults; >15 years (blue)

Having defined our s‐IgE‐positive subpopulation in terms of gender, age, and class distribution, it was now important to look at the tested food allergens individually and calculate their s‐IgE prevalence. All EAST tests that satisfied the inclusion criteria of the study were incorporated including multiple testing by the same patient (2.8% of total tests), to account for any new sensitizations appearing between tests due to possible dietary modifications or pollen sensitization. Results indicated that cow milk was the top hit (6% of the total tests analyzed), followed by cow milk, UHT (5.2%), pistachio (4.9%), soybean (4.6%), cherry (4.4%), and orange (4.4%). Lowest s‐IgE prevalence was seen for gluten (0.3%), fish mix (0.4%), olive (0.4%), cocoa bean (0.5%), and onion (0.8%). s‐IgE prevalence data for the various food allergens in all age groups combined was reported in descending order (Figure 2, black stacks). FS prevalence per age group is depicted in the same stacked bar chart using corresponding colors (Figure 2).

Top 10 food allergens found in all age groups combined were compared with those reported in each individual age group. Interestingly, this comparative approach revealed several food allergens appearing as top allergens in their respective age group but not in the analysis of all the age groups pooled together. These food items included: egg yolk in infants, meat mix (beef and lamb) and hazelnut in infants and children, peanut in infants and adults, and peach and apple in adults (Table S1).

### 3.3 IgE class distribution of top food allergens

**Figure 3:**
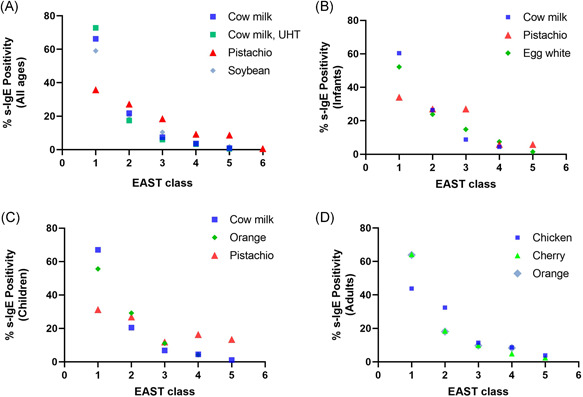
Class distribution of top food allergens. Graph indicating the specific immunoglobulin E (s‐IgE) class distribution of the top three food allergens for (A) all age groups combined: cow milk (ultra‐high temperature [UHT] in green, non‐UHT in the dark blue), pistachio (red), soybean (light blue). Graphs displaying the s‐IgE class distribution of the top three food allergens of every age group independently are also shown: cow milk, pistachio, and egg white for infants (B), cow milk, orange, and pistachio for children (C), and chicken, cherry, and orange for adults (D)

The s‐IgE class distribution for the top three food allergens of all ages combined as well as for the individual age groups was then analyzed (Figure 3). In the “all ages” graph, we noted that while approximately two‐thirds of s‐IgE detected for cow milk (including UHT) and soybean belonged to class 1, pistachio (red triangle) had a remarkably different profile. Only about a third of pistachio's s‐IgE detected belonged to class 1, while the rest was distributed between higher classes, including one case of class 6, a class that proved absent in cow milk (including UHT) and soybean allergens (Figure 3A). A similar observation was seen in the infant and children age group analysis where pistachio's distribution (red triangle) was more skewed towards higher classes unlike the rest of the top hits for the respective age groups; namely: cow milk, egg white, and orange (Figure 3B,C).

Furthermore, to shed light on the potential clinical relevance of our data, the top ten food allergens obtained in Figure 2 for all the age groups combined (comprising positive s‐IgE results from all classes 1 through 6) were compared with the top 10 food allergens identified when only the higher classes 3 to 6 were considered. Results showed that 7 out of 10 of the top food allergens (classes 1‐6) seen in Figure 2 were also seen in the list of the top food allergens (classes 3‐6) with the exception of cow milk (UHT), orange, and potato (Table S2). Interestingly, consistent with its remarkable class distribution profile, pistachio ranked first in the classes 3 to 6 top food allergen list, and the previously unseen peanut, peach, and hazelnut now ranked third, seventh, and eighth, respectively, in class 3 to 6 list (Table S2).

### 3.4 Food group sensitization ranking varies according to age group

**Figure 4:**
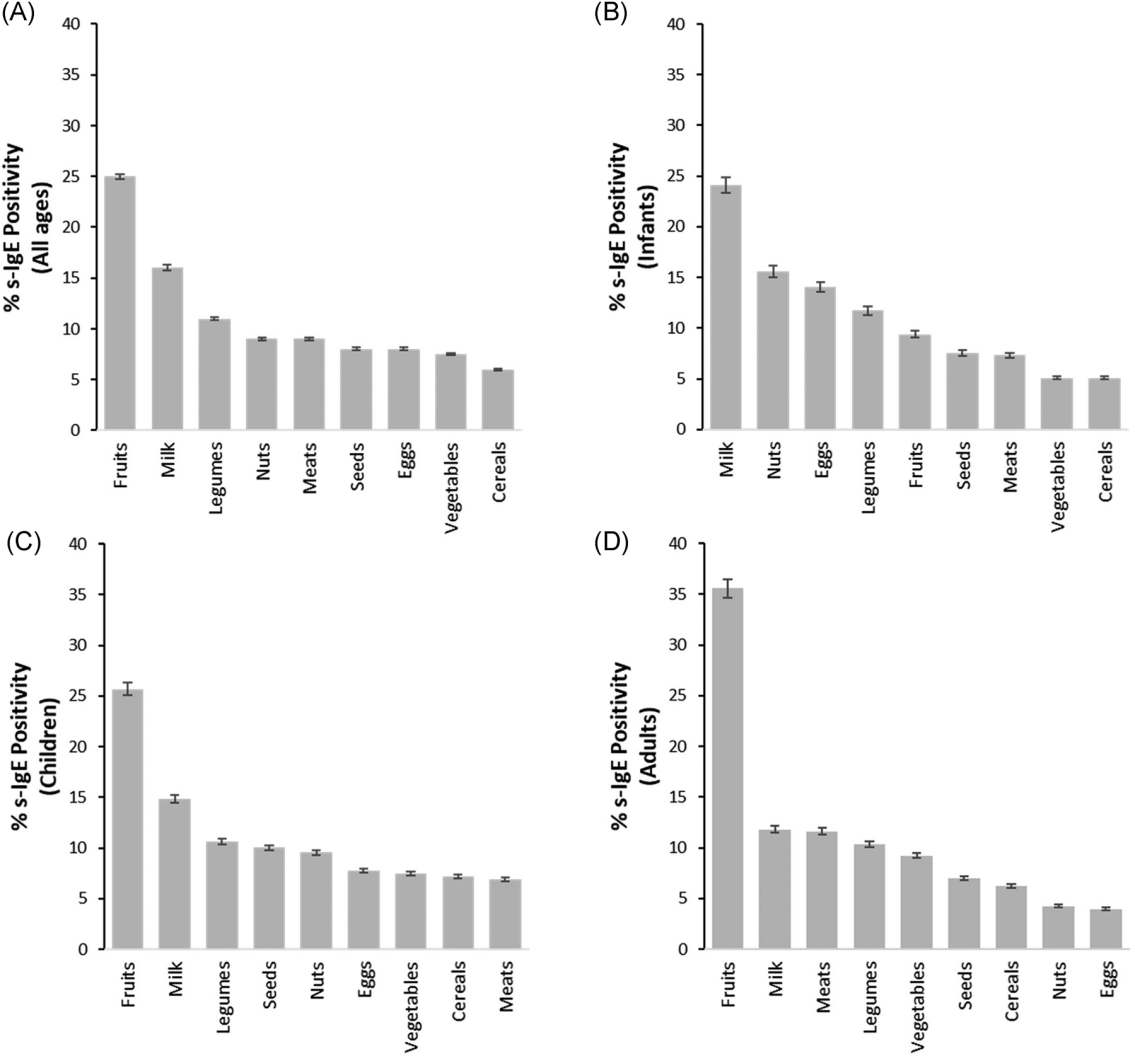
Prevalence of specific immunoglobulin E (s‐IgE) antibodies by food group. Bar graphs displaying the prevalence (%) of s‐IgE sensitization to the various food groups arranged in descending order for patients of all ages (A), infants; 0 to 2 years (B), children; 3 to 15 years (C), and adults; >15 years (D)

To achieve a broader picture of the FS results obtained, we arranged the food items found in the immunoblot panel into food groups (Table S3). Results of patients from all age groups combined revealed that the food group with the highest sensitization rates was fruits (25.3%) (top two fruits being cherry and orange) followed by milk (16%), while the lowest sensitization rates towards cereals (6.3%) and vegetables (7.5%; Figure 4A). A similar analysis was performed separately for each age group. Results showed that although the fruit group remained in the first rank for children and adults (cherry and orange remaining the top two hits for both age groups), the highest sensitization rate in infants was seen towards the milk group. The prevalence (%) of s‐IgE sensitization towards the various food groups was calculated and displayed in bar graphs for infants (Figure 4B), children (Figure 4C), and adults (Figure 4D).

## 4 DISCUSSION

Apart from a questionnaire‐based survey estimating the prevalence of FA in schoolchildren aged 6 to 14 in Al‐Karak governorate in the south of Jordan,^16^ and an FS screen of a small cohort of medically resistant chronic rhinosinusitis patients,^17^ we were unable to find any published epidemiological work about FS or FA in the country, despite an extensive literature search.

Our samples were collected in a total of 80 laboratories distributed across the country. More than 80% of these laboratories were located in the populous capital Amman, where the overwhelming majority of medical services—including allergy testing—are offered and performed. The capital is home to 42% of Jordan's population of 10.3 million as estimated in 2018 by the Jordanian Department of Statistics. Our second‐largest source of samples was the northern city of Irbid (18.5% of the population). Interestingly, these two cities alone host around two‐thirds of the total Jordanian population, making our patient cohort both relevant and representative.^18^

Patients with positive IgE antibodies against CCDs were excluded from the study since these antibodies are frequently nonspecific and often lack clinical relevance.^19^ Our analysis of 3647 food s‐IgE tests revealed that 83 tests (2.3%) were positive for CCD. This is quite low compared with values reported by other groups^19^ where a CCD prevalence rate of more than 20% was seen. The lower CCD values we obtained can be explained by the different racial/genetic background of study subjects, different food consumption patterns, different kits used for IgE testing, in addition to the fact that we report CCD prevalence to food allergens only, while the majority of studies report CCD data for food and inhalant allergens together.

Close to a quarter of our total study population was IgE‐sensitized to one or more food allergens. This finding is very similar to a report from Lebanon where 21% of patients with suspected IgE‐mediated food allergic reactions were positive for s‐IgE when tested upon their physician's request.^10^ In this study, we targeted an accessible, well‐defined, clinically relevant population namely patients suspected of food allergic reactions. It is worth mentioning that our main aim was not to assess IgE sensitization in randomly‐selected patients from the general population. Future studies that will attempt this need to be performed prospectively and will likely observe lower sensitization rates.

As far as FA is concerned, an imbalance in the direction of infant and children^20^ and female dominance^11^, ^21^ is observed in the literature. When it comes to FS, the highest prevalence is still seen in infants and children, but differences between genders become less predictable, with sensitization towards certain foods seen more in males than females.^20^ Our overall food s‐IgE results are consistent with the literature with the highest prevalence seen in infants and children (Figure 1E). In terms of gender, we observed a statistically significant correlation between gender and IgE‐mediated FS, with males having higher odds of s‐IgE positivity (Figure 1D). Several factors such as physiological and lifestyle differences could contribute to the dissimilar sensitization prevalence seen between the genders. Whether this observed gender bias is clinically significant requires further investigation.

The most commonly encountered s‐IgE antibodies for all age groups combined were against cow milk (including UTH processing), pistachio, and soybean. Interestingly, cow milk and pistachio were seen among the top three hits in infants and children but not adults, while soybean was consistently observed in the top 10 list of all three age categories (Figure 2 and Table S1). Consistent with our findings, Middle Eastern infants and children were also seen to be most commonly sensitized to cow milk in a recent study from Lebanon.^10^

Expectedly, adults exhibited a distinct sensitization profile from infants and children. The most common s‐IgE antibodies observed in adults were against chicken, cherry, and oranges. A fairly recent sensitization study from Lebanon revealed that hazelnut and wheat flower were the most common sensitizations in adults.^10^ Our adult sensitization results do not match the Lebanese study. Possible reasons could be differences in the study population's genetics, lifestyle, and dietary habits. Other potential contributing factors are differences in study design, diagnostic tools and protocols employed, and the list of food items investigated.

In terms of FA, 75% of self‐reported allergies among adults in the United States and Europe seem to involve one or more of the eight major food allergens: milk, wheat, egg, soy, peanut, tree nuts, fish and shellfish.^22^, ^23^ It is perhaps expected that sensitization data will not match self‐reported FA, especially in adults. One reason is that only a fraction of people sensitized to a particular food will go on to develop symptoms upon ingestion. Notably, a retrospective study conducted on 421 patients in Saudi Arabia revealed that while a significant proportion of patients had s‐IgE antibodies against chicken meat, only a small proportion reacted when orally challenged with the food allergen. In addition, chicken meat antibodies coexisted in the majority of study cases with hen's egg s‐IgE and rarely appeared on their own.^24^ Another potential reason why FS data do not always follow FA is that some adults will have realized what foods can cause the clinical symptoms, and will, therefore, simply stop consuming those food types for prolonged periods, yielding negative s‐IgE results.

One important finding of this study is that milk, tree nuts, and eggs represented the major food groups causing sensitization in infants, while in children and adults it was fruits followed by milk. Our results are consistent with a recent telephone call questionnaire performed on 506 Middle Eastern adult subjects to measure the prevalence of self‐reported allergy in Lebanon, which uncovered that fruits were the most common allergens seen (35%).^11^ In our study, the percentage of adult patients that developed IgE sensitization towards one or more fruits turned out to be very similar (Figure 4D). It is important to bear in mind that plant FA including fruits is more common in patients with pollinosis than the general population.^25^ This suggests that the high prevalence of cherry, orange, and peach sensitization can be due to oral allergy syndrome. Birch pollen has been shown to be associated with apple, cherry, kiwi, peach, pear, and plum oral allergies, while grass pollen with melon, orange, and peach.^26^ To elaborate with an example: two major orange allergens are Cit s 2, profilin,^27^ and Cit s 3, a lipid transfer protein (LTP).^28^ Profilins are known for their ability to cross‐react with grass pollens and LTPs with LTP allergens of other fruits such as Pru p 3 in peach.^28^ Interestingly, orange fruit allergenic extracts have also been shown to cross‐react with orange tree pollen.^29^

Several recent studies shed light on the significance of sesame seed allergy in the Middle East,^10^, ^14^ sometimes referring to it as the “Middle Eastern peanut”.^10^ Irani et al^10^ observed that while peanut allergies generally caused mild symptoms, all sesame allergy cases led to anaphylaxis. In support of these findings, our data show that sesame sensitization is seen across all age groups of our study population, surpassing peanut sensitization in children and paralleling it in adults. Sesame ranked twelfth in infants (4.6% vs 5.8% for peanut), fourth in children (7.1% vs 5.6% for peanut), and tenth in adults (1.9% vs 2% for peanut). The clinical significance of sesame sensitization in the Jordanian population and the seriousness of potential resulting allergies remain to be determined in future studies.

Very few studies explored the most commonly used tools in the diagnosis of FA in the Middle East. A Lebanese study published in 2015 found out that medical professionals mainly ordered s‐IgE blood tests (55%), followed by food elimination based on clinical history (25%), followed by skin prick testing (20%).^11^ To our knowledge, no similar studies have been published from Jordan, but given the resemblance in healthcare approaches between the two Middle Eastern countries, comparable percentages are expected.

This heavy dependence on s‐IgE testing in FA diagnosis calls for the design of customized panels that account for the particularities of the society in question. In this study, we used the EAST panel customized for nearby Turkey because all the food items it featured were popular in the Jordanian diet (with the exception of the fish mix items). This panel, however, understandably lacks some food items highly consumed in Jordan such as chickpeas, pine nuts, thyme, and certain spices. To address this, our group is currently working on generating an s‐IgE panel relevant to Jordan, building on the prevalence data obtained in this study and our knowledge of the country's dietary habits.

Despite the usefulness of our findings, this study has some limitations. The retrospective nature of this study makes it very difficult to correlate the sensitization data to patients' clinical history. It is also not possible to provide numerical data regarding the clinical manifestations which ultimately led our patients to perform the food s‐IgE tests.

Nevertheless, given that it is generally accepted that an s‐IgE class greater than 2 is interpreted as clinically significant,^30^ we decided to touch upon the potential clinical usefulness of our sensitization results by looking at the most prevalent sensitizations when only s‐IgE classes 3 to 6 were considered and compared the food items obtained with those obtained when all classes (1‐6) were analyzed (Table S2). Seven out of the top ten foods (class 1‐6) were still seen as top foods when classes 3 to 6 only were considered, this is an encouraging finding that could suggest clinical relevance of our sensitization data. Furthermore, the “class 3 to 6 approach” emphasized the important roles of tree nuts, eggs, and sesame as major allergens (pistachio ranking first, egg white second, peanut third, sesame sixth, and hazelnut eighth), while in the “class 1 to 6 approach” they ranked third, seventh, eleventh, ninth, and thirteenth, respectively). The “class 3 to 6 approach” also witnessed the fall of fruits in ranking; cherry which ranked fifth in the “class 1 to 6 approach” fell to tenth, and orange which ranked sixth did not even make it to the top 10 “class 3 to 6” list.

Finally, new studies to assess the FS and FA status in Jordan at the population level are warranted. Skin prick test results will help corroborate our s‐IgE findings, while double‐blind, placebo‐controlled food challenges are much needed to establish the prevalence of FA in Jordan. Such studies are crucial in identifying the most common food allergens taking into consideration the cultural and nutritional habits of the Jordanian people. Our study represents an important starting point for future work that can facilitate the diagnosis of food allergies in Jordan and the Middle East, and better guide patients' dietary changes and food elimination plans. This study is a significant step forward towards a better understanding of the nature and magnitude of FS in Jordan. Future studies stemming from it will undoubtedly aid in the better assessment of FA in Jordan and can help the Jordanian health authorities make informed decisions as they formulate long‐term strategic health plans concerning FA in the country. We also hope this study and similar ones will prompt the decision‐makers in Jordan to make epinephrine autoinjectors commercially available to all those whose life might depend on it.

## ETHICS STATEMENT

Ethical approval (IRB/7/2019, issued on the 11th July 2019) to conduct the study was obtained from the clinical research office of the Cell Therapy Center, University of Jordan.

## Supporting information

Supporting information

Supporting information

Supporting information

Supporting information

Supporting information

## Data Availability

The data that support the findings of this study are available from the corresponding author upon reasonable request.
