# Supplementary figures and images for: Prevalence of IgE‐mediated sensitization in patients with suspected food allergic reactions in Jordan
Source: Immun Inflamm Dis. 2020 Jun 15;8(3):384–92. doi: 10.1002/iid3.320 (PMC7416048; doi:10.1002/iid3.320)

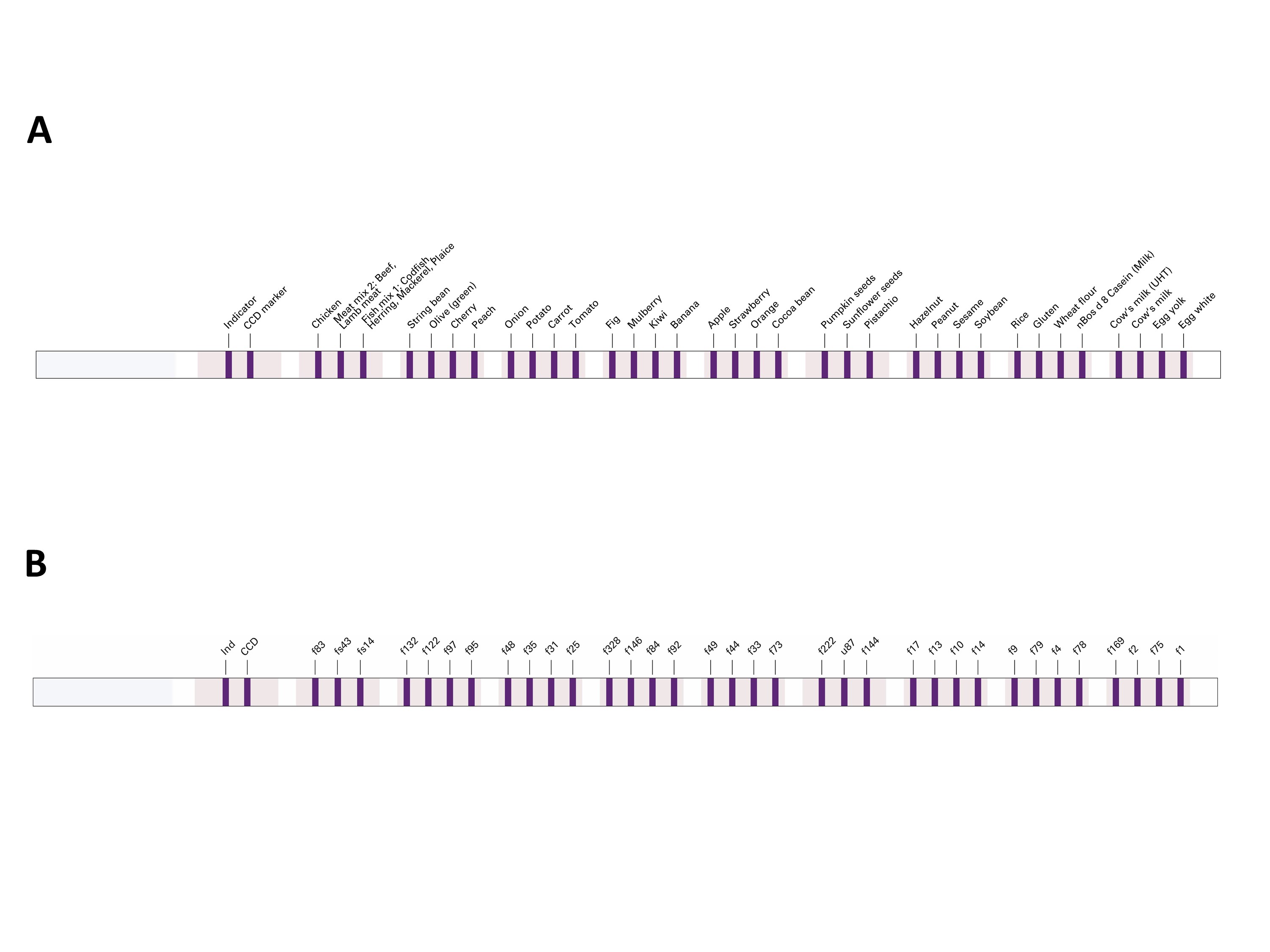

Supplement: Supplementary file 1 — Supporting information [file IID3-8-384-s001.jpg]

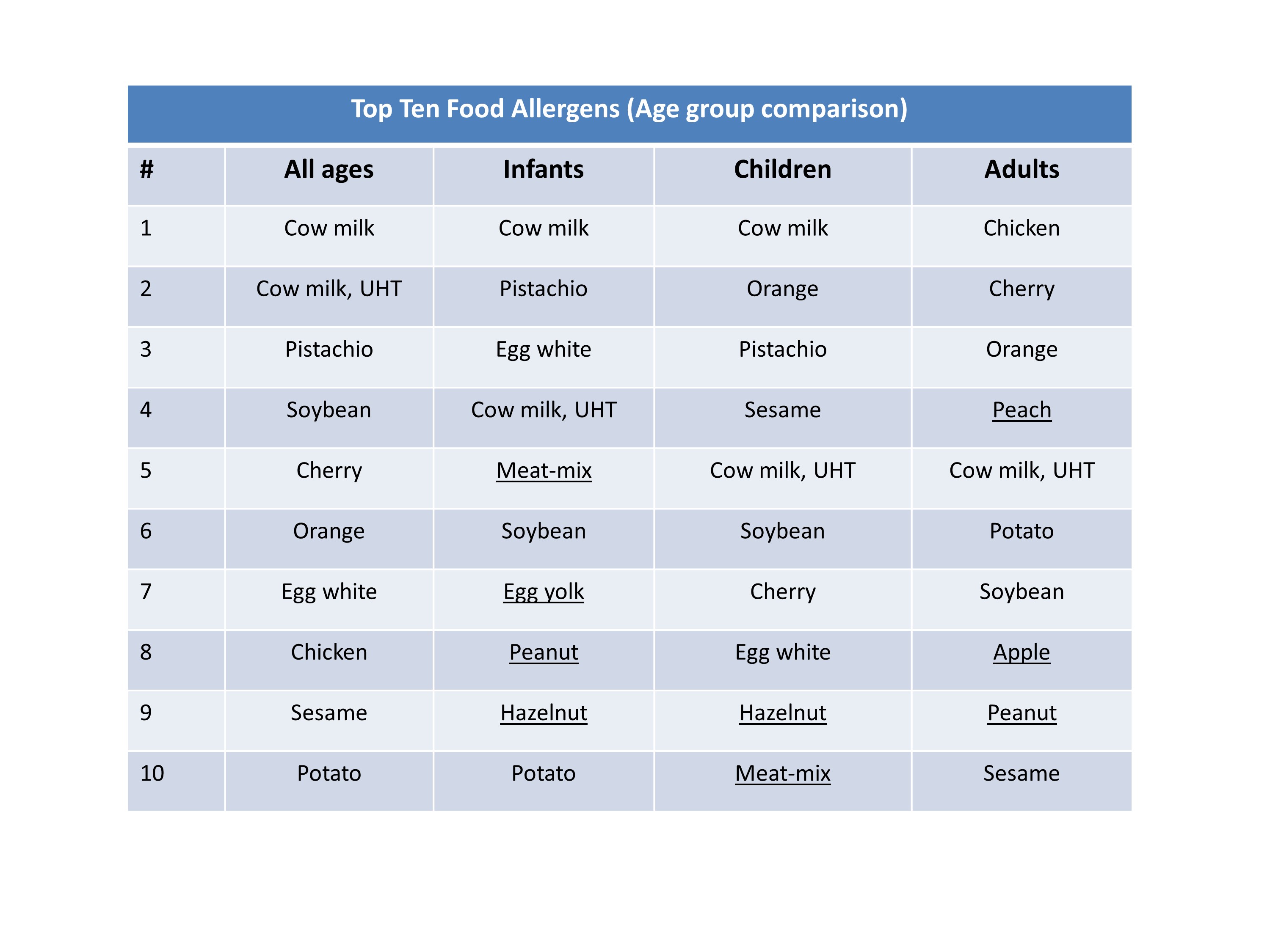

Supplement: Supplementary file 2 — Supporting information [file IID3-8-384-s002.jpg]

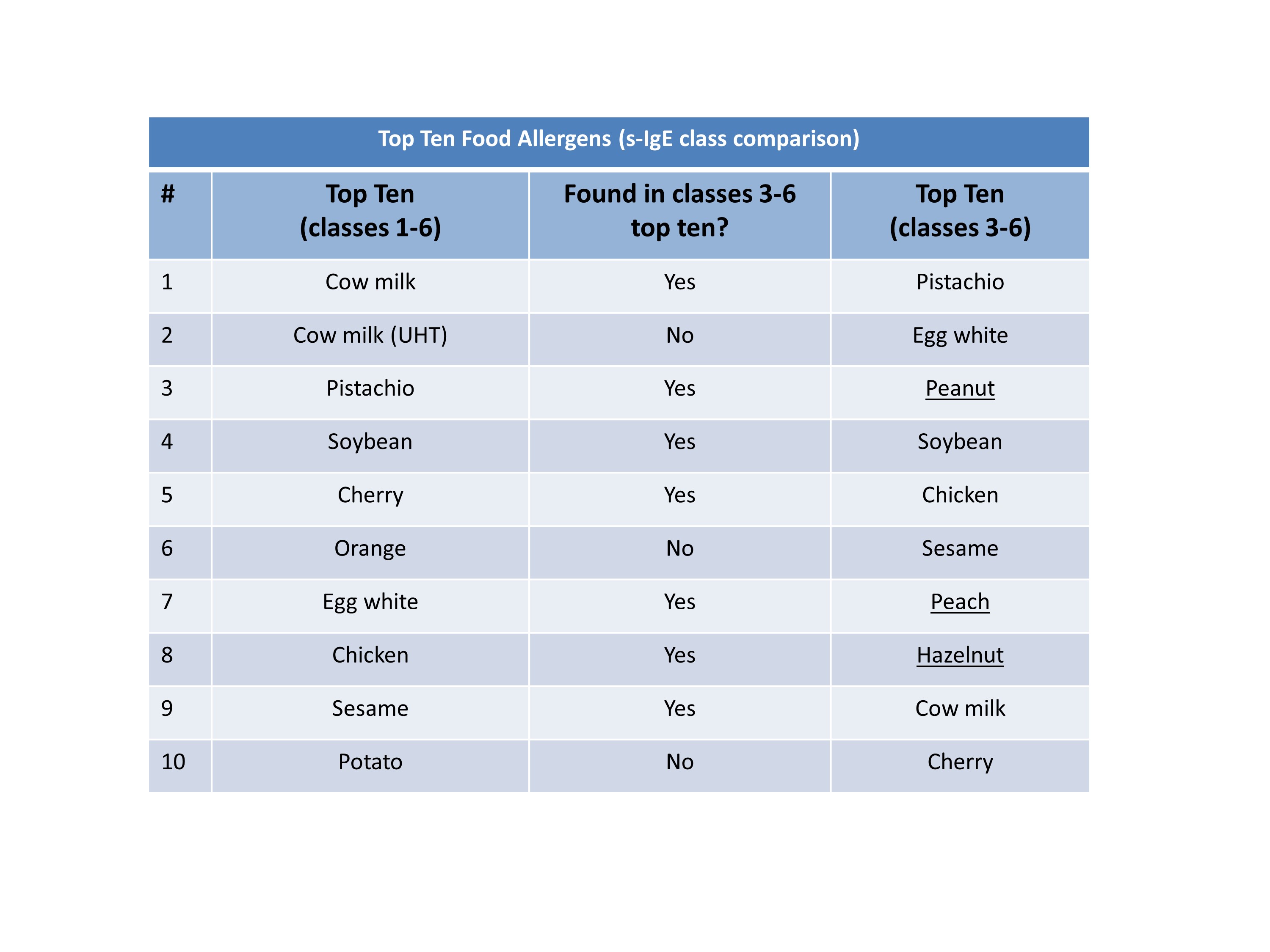

Supplement: Supplementary file 3 — Supporting information [file IID3-8-384-s003.jpg]

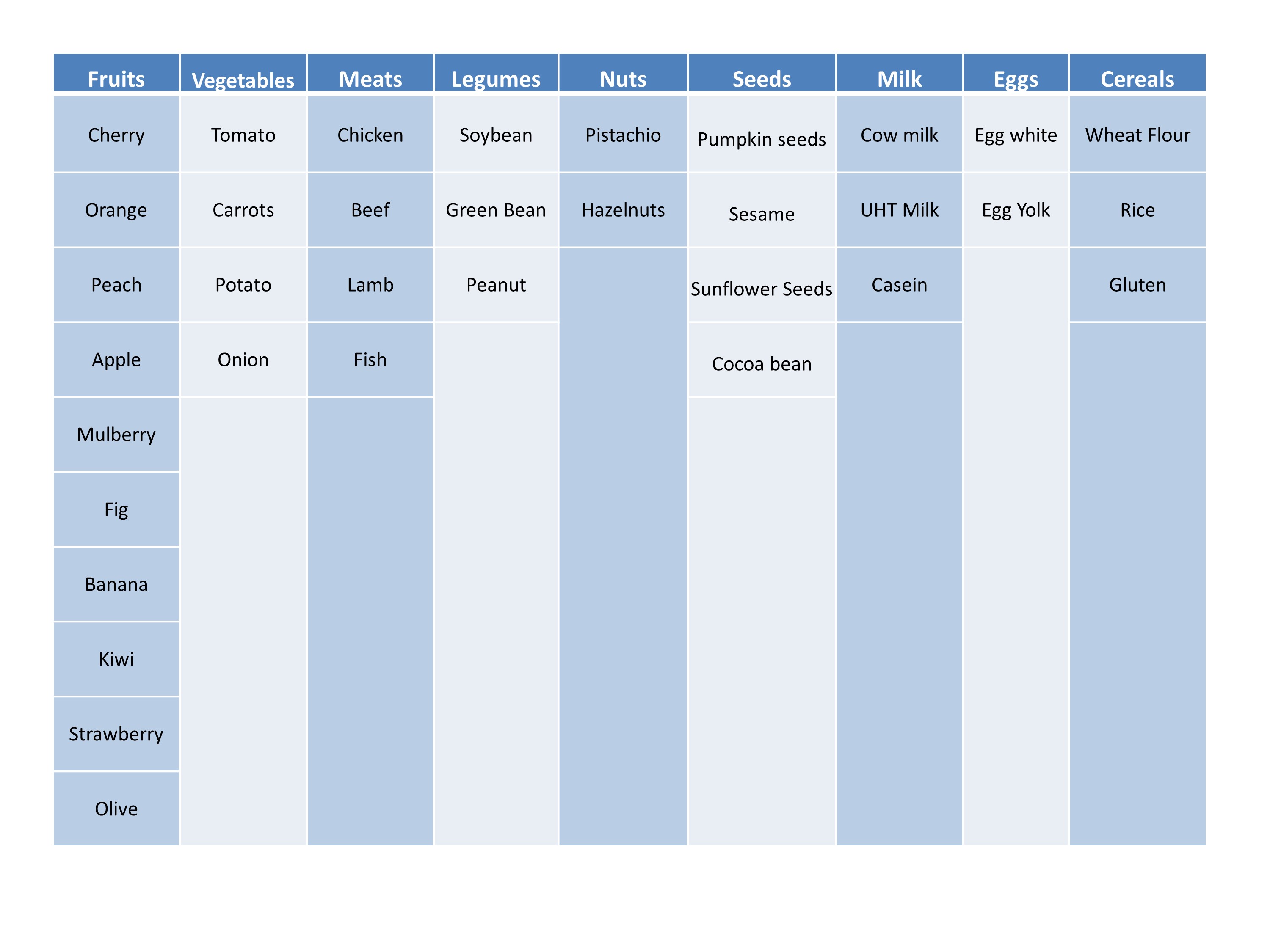

Supplement: Supplementary file 4 — Supporting information [file IID3-8-384-s004.jpg]
