# Supplementary material for: Prevalence of IgE‐mediated sensitization in patients with suspected food allergic reactions in Jordan
Source: Immun Inflamm Dis. 2020 Jun 15;8(3):384–92. doi: 10.1002/iid3.320 (PMC7416048; doi:10.1002/iid3.320)
Supplement: Supplementary file 5 — Supporting information [file IID3-8-384-s005.doc]

**Supplementary Figure 1. EUROLINE immunoblot panel.** (A) A schematic representation of the EUROLINE immunoblot food sensitization panel displaying the 34 food allergens, in addition to the CCD and indicator bands. (B) The panel in S-1A, labelled using food extract international codes instead of common names.

**Supplementary Table 1. Top ten food allergens based on age group distribution.** Table summarizing the top ten food allergens for all age groups combined, arranged in descending order by s-IgE sensitization prevalence. Findings are compared to the top ten food allergens reported per age group: Infants (0-2 Y), children (3-15 Y), and adults (>15 Y). Underlined items indicate foods found in the top ten list of any age group but not in the top ten list of all age groups combined.

**Supplementary Table 2. Top ten food allergens based on s-IgE classes.** Table summarizing top ten food allergens for all age groups combined, arranged in descending order by their s-IgE sensitization prevalence when all positive IgE classes 1-6 are included. Findings are compared to the top ten food allergens obtained when only positive s-IgE classes 3-6 are considered. Food allergens underlined in the top ten (Classes 3-6) column indicate foods not appearing in the top ten (Classes 1-6) column.

**Supplementary Table 3. Food groups composition.** A table listing the individual food items falling under each food group investigated in Figure 4.
